# X-linked SEPTIN6-related congenital neutropenia and B cell deficiency

**DOI:** 10.70962/jhi.20250173

**Published:** 2026-05-04

**Authors:** Lauren M. Gunderman, Aleksandra Petrovic, Ingrid Lundgren, Karen M. Chisholm, Sandra D. Bohling, Jonathan R. Fromm, Alexandra Ferrannini, Troy R. Torgerson, Cullen M. Dutmer, Elena W.Y. Hsieh, Safa F. Mohamad, David A. Williams, Eric J. Allenspach

**Affiliations:** 1 Center for Immunity and Immunotherapies, Seattle Children’s Research Institute, Seattle, WA, USA; 2 Department of Pediatrics, University of Washington School of Medicine, Seattle, WA, USA; 3 Pediatric Infectious Diseases, St. Luke’s Children’s Hospital, Boise, ID, USA; 4 Department of Laboratories, Seattle Children’s Hospital, Seattle, WA, USA; 5 Department of Laboratory Medicine and Pathology, University of Washington, Seattle, WA, USA; 6 Allen Institute, Seattle, WA, USA; 7 Department of Pediatrics Emory, University School of Medicine, Atlanta, GA, USA; 8 Division of Allergy and Immunology, Children’s Healthcare of Atlanta, Atlanta, GA, USA; 9 Department of Pediatrics, Section of Allergy and Immunology, University of Colorado, School of Medicine, Aurora, CO, USA; 10 Department of Immunology and Microbiology, University of Colorado, School of Medicine, Aurora, CO, USA; 11 Division of Hematology/Oncology, Department of Pediatrics, Boston Children’s Hospital, Harvard Medical School, Boston, MA, USA

## Abstract

Septins are a conserved family of hematopoietic cytoskeletal regulators. We report two full-term male siblings with a stop-loss variant in the X-linked *SEPTIN6 *gene who tested positive on SCID newborn screening and presented with myeloid tetraploidy, congenital neutropenia, absent circulating B cells, and variable T cell lymphopenia despite a normal percentage of naive T cells. In the proband, neutropenia was unresponsive to G-CSF with undetectable antineutrophil antibody. Both siblings had hypersegmented myeloid forms and tetraploidy with subsequent development of trisomy 8. One sibling had monosomy 7. Mature and progenitor B cells were markedly decreased to absent; rare plasma cells were present. Mature myeloid forms and plasma cells accumulated in the marrow leading to peripheral neutropenia and B cell lymphopenia. Matched sibling myeloablative HSCT resulted in full disease correction and minimal-to-no GVHD. In xenograft studies, altered SEPTIN6 function led to reduced early lymphoid progenitor cells, demonstrating that SEPTIN6 plays a critical role in lymphocyte development, representing a new inborn error of immunity.

## Teaser

Gunderman et al. share the second report of X-linked SEPTIN6-related disease in two brothers identified by SCID newborn screening. Findings include congenital neutropenia, absent circulating B cells, marrow mature myeloid, and rare plasma cell accumulation, as well as tetraploidy, with disease reversal following myeloablative HSCT. Xenograft studies implicate SEPTIN6 playing a role in lymphocyte development.

## Introduction

Septins are a complex, highly conserved family of GTP-binding, filament-forming proteins serving as scaffolds and diffusion barriers in various cellular processes (1). Septins form hetero-oligomeric complexes with dynamic functional filament formation (2). The 13 septins are grouped based upon amino acid sequence similarity, with one group including SEPTIN6, SEPTIN8, SEPTIN10, SEPTIN11, and SEPTIN14 (1). The SEPTIN2/6/7 complex demonstrated GTP-binding activity with a role in regulating cytokinesis (3, 4). While the role of SEPTIN6 in human lymphoid development is unknown, several previous findings point to a role of SEPTIN6 in human myeloid development. Renella et al. recently identified a germline de novo stop-loss mutation in *SEPTIN6*, previously known as SEPT6, in a male infant with severe G-CSF refractory neutropenia and progressive dysmyelopoiesis with tetraploid myeloid precursors, reversed by hematopoietic stem cell transplantation (HSCT) (5). Somatic septin fusion partners, particularly with the KMT2A gene (also known as MLL), have been implicated in cases of infant and early childhood acute myeloid leukemia (6, 7).

Lymphocytes develop from long-term hematopoietic stem cells (HSCs) in the bone marrow (BM) progressing through intermediate progenitors including multipotent progenitors to become more lineage-restricted, lymphoid-biased (LMPP) progenitors (8). These specialized intermediates start to express interleukin-7 receptor α (IL-7Rα, also known as CD127) en route to the common lymphoid progenitor (CLP), yet retain some developmental flexibility. IL-7Rα is required for T cell development and deficiency results in a severe combined immunodeficiency (SCID) phenotype. In contrast, B and natural killer (NK) cell development does not require IL-7 in humans. Instead, IL-7 signaling induces proliferation of early B lymphocyte progenitors and increases the expression of EBF1 and PAX5 proteins, which are necessary for B cell specification and commitment while further limiting myeloid potential (9). While neutropenia is not uncommon in a SCID phenotype, this is often a secondary and transient process with the notable exception of AK2 (10) and RAC2-related SCID patients (11). Neutropenia can also be observed in B cell deficiencies such as X-linked agammaglobulinemia, although often transient during infectious complications and rarely observed after initiation of immunoglobulin replacement (12). The mechanism of neutropenia in B cell deficiencies remains unclear.

Here, we report two male siblings with severe nonsyndromic congenital neutropenia with diverse myeloid forms demonstrating dysplastic nuclei including hypersegmentation. The siblings had positive SCID newborn screening (NBS), absent circulating B cells, and tetraploid karyotype. Both children harbored a maternally inherited stop-loss variant in the X-linked *SEPTIN6 *gene and subsequently were treated with a matched related HSCT. Herein, we discuss these clinical cases and provide functional data regarding lymphoid development in SEPTIN6-related disease.

## Results

### Two affected siblings presented with severe congenital neutropenia and absent circulating B cells following positive SCID NBS

**Figure 1.**
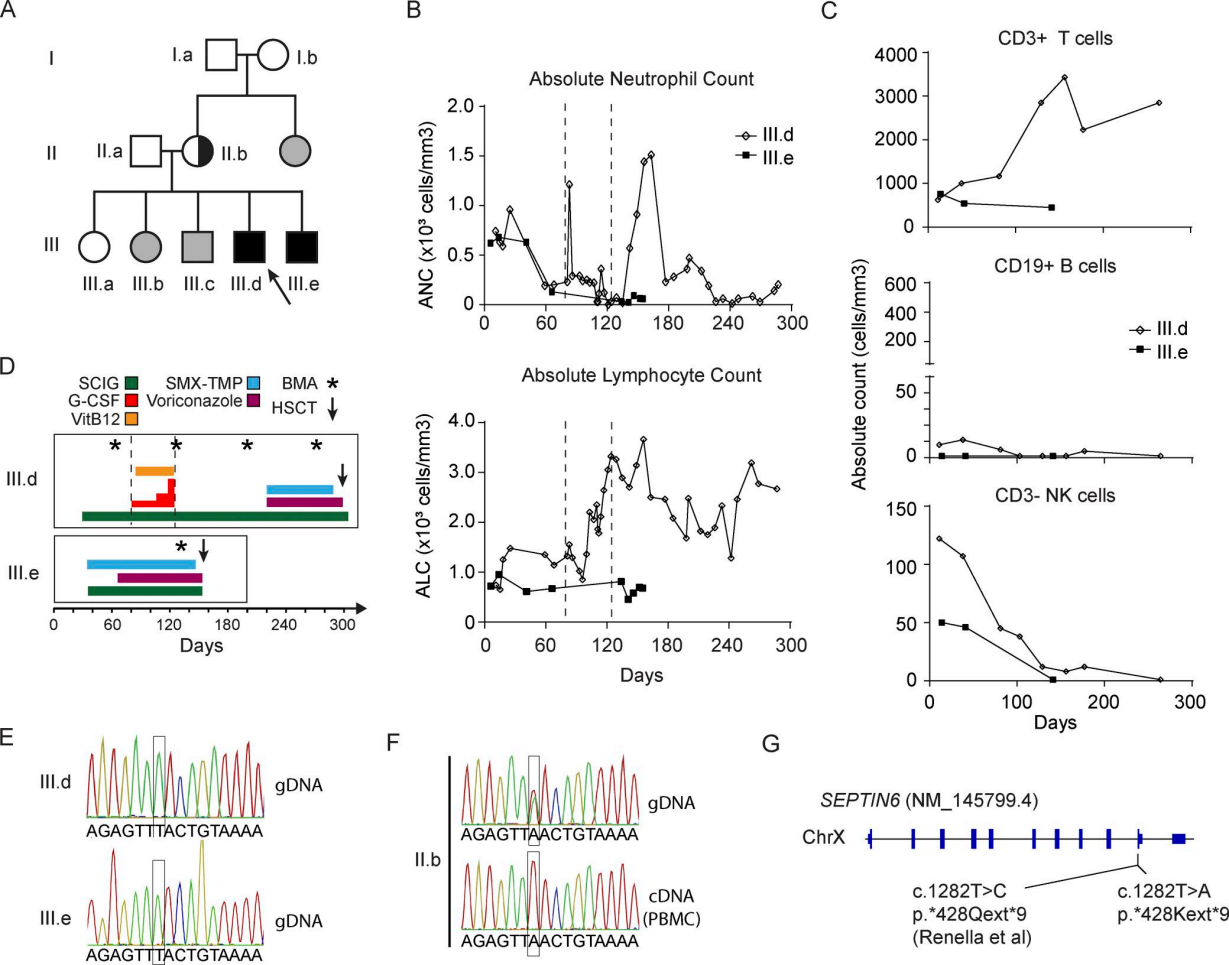
**Clinical course of two affected siblings with X-linked SEPTIN6 stop-loss variants. (A)** Three-generation family pedigree demonstrates proband (III.d; arrow) and affected sibling (III.e) with congenital neutropenia, dysmyelopoiesis with tetraploidy and aneuploidy, B cell aplasia, and variable T cell lymphopenia. Solid black squares are affected males. Unaffected individuals are either SEPTIN6 WT (white shapes) or not genotyped (gray). The sibling’s mother was an asymptomatic carrier. **(B)** ANC and ALC were enumerated for III.d and III.e. The period of G-CSF treatment for III.d is indicated via vertical dotted lines. **(C)** Serial lymphocyte subsets enumerated CD3^+^ T cells (top), CD19^+^ B cells (middle), and CD3^−^CD16^+^ and/or CD56^+^ NK cells (bottom) over the clinical course for III.d and III.e, respectively. **(D)** Clinical course pretransplant for the proband (III.d) and affected sibling (III.e) following the initial positive SCID NBS including initiation of weekly SCIG (green), SMX-TMP (blue) and voriconazole (purple) prophylaxis, vitamin B12 (orange), and G-CSF (red; 5 μg/kg/dose is 1 bar; max 20 μg/kg/dose). BMA/BM biopsies (asterisk) were collected as indicated. **(E and F)** Sanger sequencing of genomic DNA isolated from PBMCs from mother (II.b), proband (III.d), and affected sibling (III.e) identifies the variant (GRCh38 ChrX:119625378A>T) within *SEPTIN6* exon 10 (NM_145799.4). **(F)** Sanger sequencing of complementary DNA (cDNA) converted from bulk PBMC mRNA from the mother (II.b) demonstrated only the WT allele was present. **(G)** Diagram of *SEPTIN6* genetic locus highlighting the current identified mutation compared with the previously known mutation (5). The same neopeptide (LCCLLHAA*) extension is predicted to occur in both stop-loss variants. SCIG, subcutaneous immunoglobulin; SMX-TMP, sulfamethoxazole/trimethoprim; BMA, bone marrow aspirates.

Two nonsyndromic male siblings (III.d and III.e) born 4 years apart to nonconsanguineous parents developed congenital neutropenia and absent B cells after testing positive for SCID NBS (Fig. 1 A). Proband (III.d) was delivered at term with a birthweight of 3,359 g following an uncomplicated pregnancy. Family history was noncontributory. An abnormal SCID NBS showed (T-cell receptor excision circles (TRECs) of 9 copies (normal >20), and confirmatory blood testing at 11 days old revealed leukopenia (WBC 1,840 cells/mm^3^) with an absolute neutrophil count (ANC) of 740 cells/mm^3^ and an absolute lymphocyte count (ALC) of 740 cells/mm^3^ (Fig. 1 B). No infectious symptoms were present. A peripheral blood smear was negative for blasts but showed neutrophils with dysplastic nuclei, some of which were hypersegmented. Lymphocyte subset testing showed T and B cell lymphopenia (absolute counts: CD3^+^ T cells 625 cells/mm^3^ with a CD4:CD8 ratio of 1.6; CD19^+^ B cells 8 cells/mm^3^; CD3^−^CD56^+^ NK cells 63 cells/mm^3^) (Fig. 1 C), confirmed the same day at a different laboratory (CD19^+^ of 2 cells/mm^3^ and CD3^+^ of 548 cells/mm^3^), and the T cells were 90% naive (CD45RA^+^). Repeat TREC testing (different laboratory) remained low at 1,835 copies (normal >/=6,794). Erythrocyte adenosine deaminase (ADA) activity was low (46% of controls), but deoxyadenosine nucleotides were undetectable, thus not consistent with ADA-SCID. At 4 wk of age, lymphocyte subsets showed improved CD3^+^ T cell counts of 1,004 cells/mm^3^ with a CD4:CD8 ratio of 2.7 and 90% CD45RA^+^. The B cells remained low with 11 CD19^+^ cells/mm^3^. NK cells were normal with 107 CD56^+^ cells/mm^3^. Repeat TREC counts normalized at 36.7 copies (normal >20), and proliferative responses to phytohemagglutinin (PHA) and pokeweed mitogens were normal. Overall, testing was not consistent with SCID. Early standard genetic testing for the SCID workup was negative including a 407-gene sequencing panel for primary immunodeficiency diseases and chromosomal SNP microarray. Given the sustained B cell defect and neutropenia, subcutaneous (SC) immunoglobulin and prophylaxis with voriconazole and sulfamethoxazole/trimethoprim were initiated during further workup (Fig. 1 D).

The proband’s younger brother (III.e) developed similar initial symptoms. He was born at 36 wk via cesarean section due to placenta previa but had no perinatal complications. A cord blood sample was tested given his recent family history, which demonstrated WBC of 5,300 cells/mm^3^ with an ANC of 4,346 cells/mm^3^ and an ALC of 650 cells/mm^3^. An abnormal SCID NBS resulted in TREC counts of zero (normal >20 copies). Lymphocyte subset testing on the first day of life showed T cell lymphopenia (CD3^+^ of 710 cells/mm^3^) and absent CD19^+^ B cells (Fig. 1 C). At 7 days old, he had leukopenia (WBC of 1,600 cells/mm^3^) with an ANC of 620 cells/mm^3^ and an ALC of 720 cells/mm^3^ (Fig. 1 B). At 4 wks of life, repeat lymphocyte subsets showed sustained B cell deficiency (CD19 ^+^ 1 cell/mm^3^) and ongoing T cell lymphopenia (CD3^+^ 760 cells/mm^3^), although mostly naive phenotype (CD45RA^+^ 95%). NK cells were normal (50 cells/mm^3^). Repeat TRECs at another laboratory were moderately reduced at 4,075 (normal >6,794 copies) despite improved T cell counts. (At the time of draw, lymphocytes showed CD3^+^ T cells 451 cells/mm^3^, CD4^+^ T cells 219 cells/mm^3^, CD8^+^ T cells 237 cells/mm^3^, and CD19^+^ B cells <1%.) His lymphocyte proliferative responses to PHA were normal. Given concern for a similar disease process, he was started on weekly SC immunoglobulin, and sulfamethoxazole/trimethoprim prophylaxis, and was placed in home isolation (Fig. 1 D).

### Maternally inherited SEPTIN6 stop-loss variant shows negative selection

To further investigate the molecular basis of this phenotype, whole-genome sequencing in the proband identified an X-linked maternally inherited likely pathogenic stop-loss variant in *SEPTIN6* (NM_145799.4) c.1282T>A; p.*428Kext*9 (NP_665798.1) (Fig. 1 E). Targeted familial variant genetic testing confirmed the presence of the same mutation in the younger brother (III.e). The mother (II.b) was an asymptomatic heterozygous carrier of the variant, and maternal grandparents (I.a and I.b) and eldest sister (III.a) tested negative. Next, we sequenced gDNA and complementary DNA (cDNA) from the same maternal blood sample and found monoallelic expression only of the unmutated *SEPTIN6* allele suggesting a strong negative selection for the variant during hematopoiesis (Fig. 1 F). Overall, the SEPTIN6 stop-loss variant segregated with disease demonstrating an X-linked recessive inheritance in this family. Of note, a remarkably similar case of X-linked congenital neutropenia was previously reported with a de novo stop-loss SEPTIN6 variant (c.1282T>C; p.*428Qext*9) to an alternate amino acid (Fig. 1 G) (5). Both stop-loss variants result in a similar neopeptide (LCCLLHAA*) extension. In contrast to the previous report, no somatic genetic rescue events were identified.

### G-CSF refractory congenital neutropenia in SEPTIN6-related disease

Congenital neutropenia was a prominent disease feature shared by the affected siblings (III.d and III.e) and was also described in the previous case of the SEPTIN6-related disease (Fig. 1 B) (5). The proband (III.d) had considerable diagnostic evaluation prior to his genetic diagnosis. Antineutrophil antibodies were absent. Fanconi anemia, chromosomal breakage study, and telomere length testing were normal. A peripheral blood smear from III.d showed decreased neutrophils at 8 wk of age, including some with multisegmented nuclei. Testing for metabolic defects showed normal folate of 14.4 ng/ml (reference >3.0 ng/ml), vitamin B12 of 369 pg/ml (reference 239–931 pg/ml), and homocysteine of 10.20 µmol/L (reference 6.6–14.8 µmol/L). The methylmalonic acid serum level was mildly increased at 1.46 µmol/L (reference 0–0.4 µmol/L). Treatments as outlined in Fig. 1 D including weekly vitamin B12 did not change blood counts. G-CSF was started for III.d at 5 μg/kg/dose twice weekly eliciting a transient response (ANC of 1,210 cells/mm^3^) 2 days following the first dose but quickly dropping again with severe neutropenia (ANC of 290 cells/mm^3^) only days later. The G-CSF dose was escalated to 10 μg/kg/day and then 10 days later to 20 μg/kg/day without improvement in the neutropenia. Given lack of response, G-CSF was discontinued. Several weeks after stopping G-CSF therapy, the neutropenia transiently improved (Fig. 1 B). Viral PCR testing of the serum was negative for CMV, EBV, parvovirus, and HHV6. The ANC peaked at 1,510 cells/mm^3^ in the setting of WBC 4,300 cells/mm^3^, Hgb 10.2 g/dl, platelet count 489,000 cells/mm^3^, and ALC 2,500 cells/mm^3^. However, the following week severe neutropenia returned (ANC of 230 cells/mm^3^), which then persisted. Interestingly, the lymphopenia improved during G-CSF dose escalation and remained in the normal range long after discontinuation. In contrast, the younger affected brother (III.e) demonstrated more persistent cytopenias including persistent neutropenia and T cell lymphopenia (Fig. 1, B and C). Whether this difference in T cell lymphopenia between siblings is due to G-CSF treatment is unclear.

### Dysmyelopoiesis and cytogenetic abnormalities in SEPTIN6-related disease

**Figure 2.**
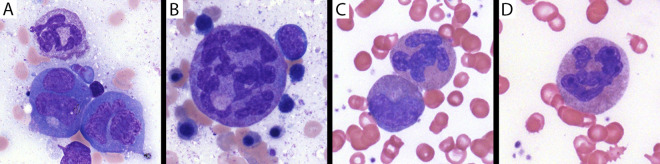
**Dysmyelopoiesis in the BM of patient III.d with SEPTIN6-related disease. (A)** Mature neutrophil and two binucleate promyelocytes in the BM collected at 4 mo of age (after G-CSF). **(B)** Markedly enlarged mature neutrophil with hypersegmentation and surrounding erythroid precursors in the BM collected at 4 mo of age (after G-CSF). **(C)** Mature neutrophil with abnormal lobation and metamyelocyte in the BM aspirate collected at 9 mo of age. **(D)** Mature neutrophil with abnormal lobation in the BM aspirate collected at 9 mo of age. (Wright–Giemsa stains, 1,000× magnification).

**Figure S1.**
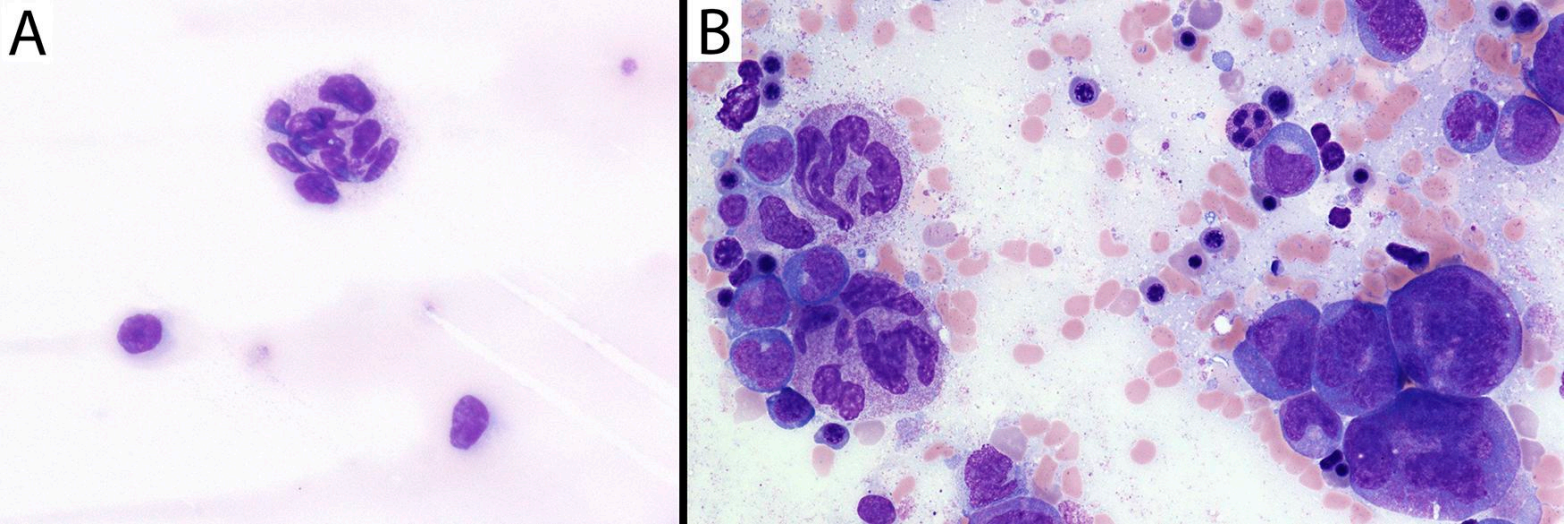
**Dysmyelopoiesis in peripheral blood and BM of patient III.d at 4 mo of age. (A)** While neutrophils in the peripheral blood were markedly decreased (ANC of 190 cells/mm^3^), rare forms at the feathered edge were enlarged and had increased nuclear lobes (Wright–Giemsa stain, 400× magnification). **(B)** BM aspirate at the same time showed left-shifted but full-spectrum maturation. Many myeloid precursors were enlarged and had increased nuclear lobation at all stages of maturation, but rare morphologically normal forms are present (mid-upper right) (Wright–Giemsa stain, 400× magnification).

**Figure S2.**
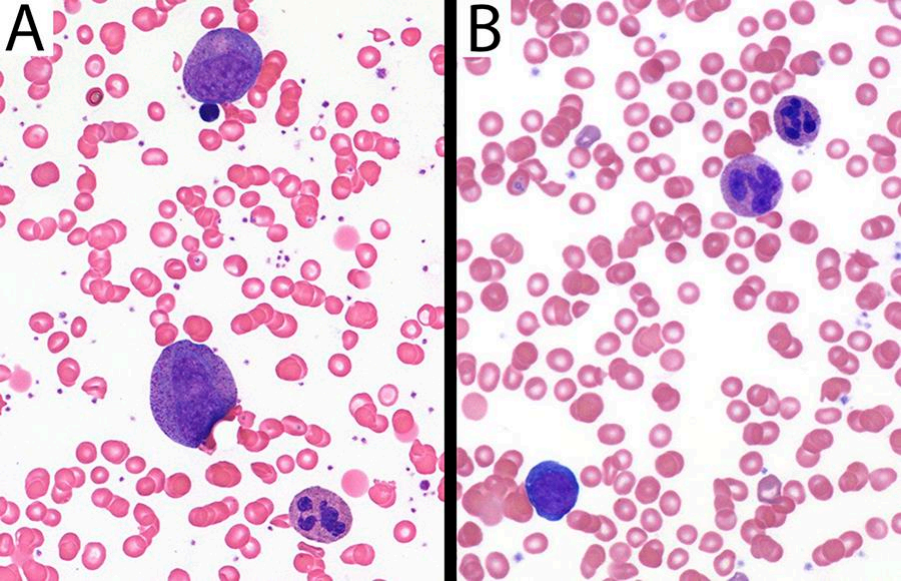
**Presence of morphologically normal neutrophils in the BM. (A)** Mature neutrophil and two enlarged myeloid precursors in patient III.d in the BM performed at 9 mo of age. **(B)** Mature neutrophil, abnormally lobated neutrophil, and early erythroid precursor in the BM performed in patient III.e at 4 mo of age.

Siblings III.d and III.e demonstrated progressive dysmyelopoiesis with tetraploid myeloid precursors and a predisposition to aneuploidy similar to the previously reported SEPTIN6 stop-loss case (5). Proband III.d underwent serial BM aspirates and core biopsies, while the younger sibling (III.e) only required one BM aspirate and biopsy pretransplantation (details in Tables S1 and S2). Across these samples, BM biopsies consistently showed hypersegmentation of fully matured neutrophils based upon morphology (Fig. 2, A–D; Fig. S1 B; and Fig. S2, A and B), which was also visible in a majority of mature neutrophil forms in peripheral blood as shown for III.d (Fig. S1 A). Myeloid precursors were enlarged with increased nuclear lobation (Fig. S1 B), but rare morphologically normal forms were also present (Fig. 2, C and D; and Fig. S2, A and B). No myeloid maturation arrest was appreciated. Reciprocal monocytosis during the neutropenia was also not present (in either sibling). While a few neutrophils had long, thin chromatin filaments between nuclear lobes, no vacuoles were observed. Overall, there was visualization of full-spectrum maturation of neutrophils in both the peripheral blood and BM of the siblings, as well as rare morphologically normal forms without hypersegmentation. Several premalignant somatic aneuploidies (trisomies of chromosomes 7, 8, and 9) had been reported in the previously reported case of SEPTIN6-related disease (5). Similarly, cytogenetic analysis was consistently demonstrated with a tetraploid karyotype (∼30–50% of cells) in all samples analyzed. Furthermore, somatic trisomy 8 was observed in a subpopulation of BM cells in both infants and monosomy 7 in the younger sibling (patient III.e), which was surprisingly already present at the initial BM biopsy (4 mo of age), highlighting the predisposition to aneuploidy. Early BM biopsies (2 and 4 mo) were normocellular; however, at 9 mo the proband (III.d) demonstrated hypocellular marrow for age (∼50%) with reduced myeloid precursors and megakaryocytes. In the Renella et al. paper, the BM samples at 3 and 6 mo demonstrated hypocellularity in the infant affected with SEPTIN6-related disease (5). Thus, the cellularity of the BM may be more variable.

### Abnormal B cell lymphopoiesis despite the presence of plasma cells

**Figure 3.**
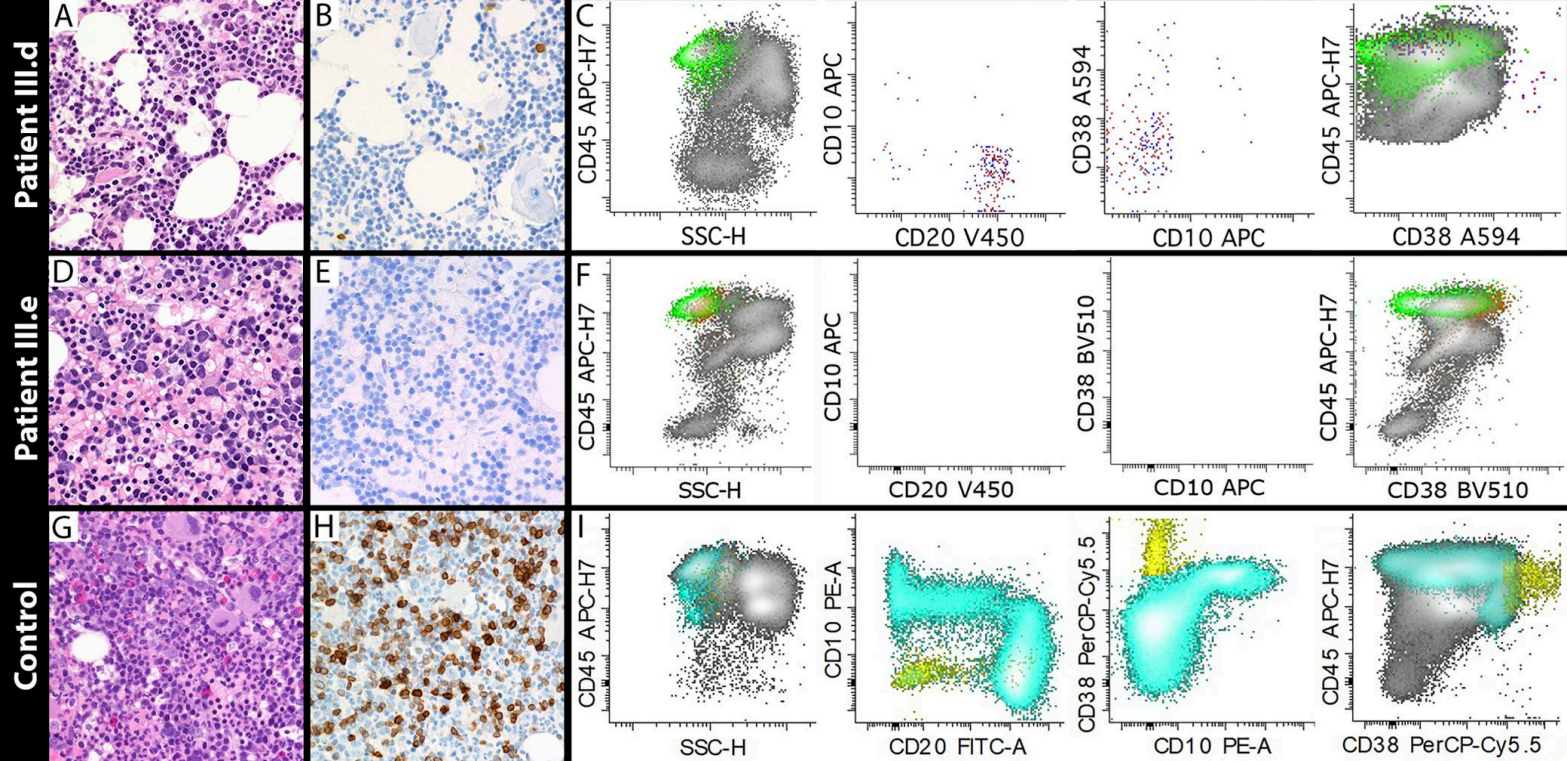
**Rare-to-absent mature B cells/plasma cells in the BM of patients with SEPTIN6-related disease compared with the age-matched control. (A)** H&E-stained section of BM biopsy from patient III.d collected at 9 mo of age (hypocellular with ∼50% cellularity). **(B)** Rare plasma cells are seen morphologically and highlighted with CD79a immunostain. **(C)** BM flow cytometry results showing rare mature B cells with polytypic surface light chain expression (representing 0.15% of nonerythroid cells) and rare plasma cells (with bright CD38 expression in the fourth plot, representing 0.004% of nonerythroid cells). First and fourth plots include all viable events; second and third plots depict CD19^+^ events; red = positive for surface κ light chain; blue = positive for surface λ light chain; green = mature T cells. **(D)** H&E-stained section of BM biopsy from patient III.e collected at 2 mo of age (normocellular with 90–95% cellularity). **(E)** Lack of plasma cells or B cells based upon CD79a staining. **(F)** BM flow cytometry results showing essentially absent B cells and plasma cells (color scheme same as panel C). **(G)** 9-mo-old age-matched infant being worked up for anemia of unclear etiology. H&E-stained section of BM shows normocellular marrow for age (>90%). **(H)** CD79a immunostain in the age-matched control. **(I)** Flow cytometry results from an age-matched control showing abundant CD19^+^ B cells including maturing hematogones (CD10^+^, teal), mature B cells (CD20^+^, teal), and plasma cells (bright CD38, yellow). Note: Histology photomicrographs at 400× magnification.

B cell lymphopenia was another persistent finding across affected individuals with SEPTIN6-related disease. In the previously reported SEPTIN6 stop-loss case, a BM sample at 3 mo showed a similar paucity of B cells (<0.5% of cells) and plasma cells (5). In that reported case, at 6 mo, the BM demonstrated <1% CD19^+^ B cells and a mixture of mature λ and κ positive cells, similar to our findings. To determine whether there was a block in B cell development, we performed analysis of BM samples including immunohistochemistry with CD79a, a pan-B cell marker from early B cell progenitors to plasma cells. At 9 mo of age, the BM demonstrated a paucity of CD79a-positive cells (<1% of cells), consistent with the low B cells in circulation. These rare CD79a^+^ cells had the morphology of plasma cells (Fig. 3, A and B). By flow cytometry, there was a striking reduction in hematogone (CD19^+^CD10^+^) cells. However, rare populations of mature B cells (CD19^+^CD20^+^CD10^−^CD38^−^) with polytypic light chain expression and plasma cells (CD20^−^CD10^−^CD38^bright^) were present (Fig. 3 C). In the younger affected sibling (III.e), BM collected at 2 mo of age had virtually no CD79a staining (Fig. 3, D and E) and flow cytometry demonstrated almost complete absence of mature B cells or progenitors including CD19^+^CD10^+^ cells (Fig. 3 F). For comparison, diffuse CD79a staining was found in a BM sample from an age-matched control (9 mo old) evaluated for anemia (Fig. 3, G and H). Flow cytometry showed typical abundant CD19^+^ B cells including maturing hematogones (CD10^+^, teal), mature B cells (CD20^+^, teal), and plasma cells (bright CD38, yellow) (Fig. 3 I). Reduction in early B cell progenitors has been observed in settings of high interferon (IFN) expression, but IFNs α, β, and γ were all low to undetectable in the serum of our patients (Table S3). These data suggest early B cell progenitors are reduced in SEPTIN6-related disease.

Despite the low B cell numbers, we found evidence that antibody-secreting cells could develop in SEPTIN6-related disease. The proband (III.d) showed elevated IgM levels starting at 4 mo at 312 mg/dl (normal 25–100 mg/dl), peaking at 999 mg/dl at 5 mo of age, and then steadily dropping to 123 mg/dl at 7 mo just prior to HSCT. The spike in IgM correlated with the transient recovery of neutrophils a few weeks after stopping G-CSF therapy, but CD19^+^ B cells were not detectable. Infectious disease screening was negative, and CD40 ligand expression was normal. In contrast, his younger brother (III.e) had low antibody levels with IgM 13 mg/dl (normal 27–132 mg/dl) at 6 mo of age prior to transplantation. Due to the observed IgM spike, we reasoned that rare B cells in circulation could differentiate into plasma cells in the setting of the SEPTIN6 mutation, which was also supported by our BM findings.

**Figure 4.**
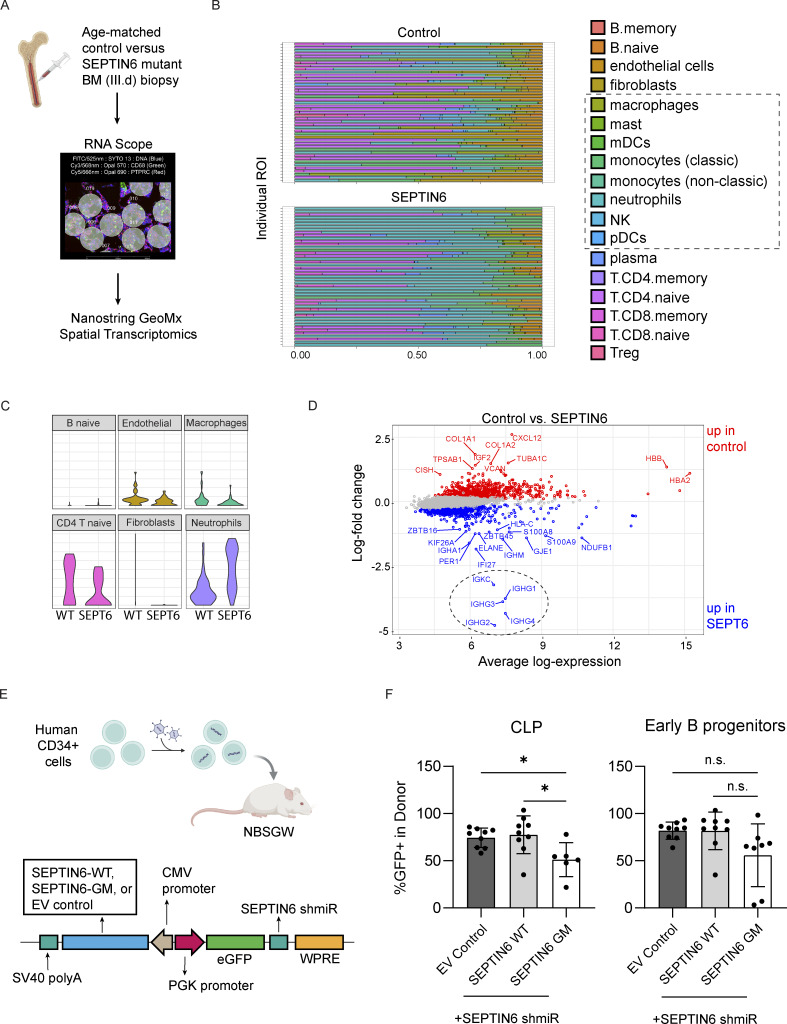
**Mutant SEPTIN6 BM tissue has plasma cells and mature neutrophils, despite reduced early lymphoid progenitors. (A)** Diagram of workflow for mRNA transcriptional analysis from pooled digital spatial transcriptomics (DSP) data acquired from FFPE tissue sections prepared from BM biopsy #3 from our proband (III.d) versus age- and gender-matched control. **(B and C)** (B) Cellular deconvolution estimates for the proportions of different cell types in each ROI were performed for each sample, and (C) the major populations are summarized. **(D)** Pooled regions (*n* = 49 ROIs per subject) were analyzed for gene expression and log fold change by log expression plotted to compare the SEPTIN6 mutant versus control marrow. The dotted circle highlights genes with high expression in plasma cells. **(E)** Human CD34^+^ cells were transduced with lentivirus, and 2 days after transduction, 1 million cells were retro-orbitally injected into NBSGW mice. Diagram of the bidirectional LV to express a WT or GM SEPTIN6 (*428Qext*9) protein (5) or empty vector. Transduced cells express an eGFP reporter cis-linked with shmiR to restrict endogenous SEPTIN6 expression compared with an empty vector control. **(F)** BM was isolated at 4 mo after transfer and the percent GFP+ determined by flow cytometry for each progenitor population. CLPs were gated Lin^−^CD34^+^CD38^+^CD19^−^CD10^+^ CD45RA+CD135^−^ (left), and early B cell progenitors were gated Lin^−^CD34^+^CD38^+^CD19^+^CD10^+^ (right). Results are pooled animals from 7 human donors across two experiments. *P < 0.05, unpaired student *t* test. A schematic was generated in BioRender. EV, empty vector; GM, genetic mutant; shmiR, short hairpin miRNA. (mDC: myeloid dendritic cells; pDC: plasmacytoid dendritic cell; Treg: regulatory T cell; WPRE: Woodchuck Hepatitis Virus Posttranslational Regulatory Element).

To more broadly assess the cell composition including plasma cells in the BM of SEPTIN6-related disease, we performed spatial transcriptomics on a BM biopsy from the proband (III.d) at 7 mo of age and compared it with an age- and gender-matched healthy donor control sample (Fig. 4 A). We used in situ hybridization probes to select equal regions of interest (ROI) with similar cell counts prior to incubating with a whole transcriptome UV-cleavable probe library. Cellular deconvolution is a technique that estimates the proportions of different cell types in samples collected across unique ROIs. Using this technique, we found a consistent reduction in lymphocytes, both T and B cells, in the BM from III.d and increased myeloid populations compared with the control BM based upon transcriptional data (Fig. 4, B and C). Pooling the ROI for each individual showed III.d had an increased expression of B cell and plasma cell–specific genes (*IGHG1*, *IGHG2*, *IGHG3*, *IGHG4*, and *IGKC*) compared with the control sample, but lower evidence of naive B cells (Fig. 4 D). Furthermore, genes including those associated with granulocyte development (*ELANE*, *S100A8*, *S100A9)* were elevated in the SEPTIN6-related disease compared with the healthy controls. These data suggest myeloid-skewed BM populations while we see reduced lymphoid cells. Although limited from the single case (III.d) versus control comparison, these findings highlight that SEPTIN6-related disease supports plasma cell development despite significant reduction in circulating naive B cells in the BM and peripheral circulation.

### SEPTIN6 variant limits early lymphoid progenitors reducing mature B and plasma cells

**Figure S3.**
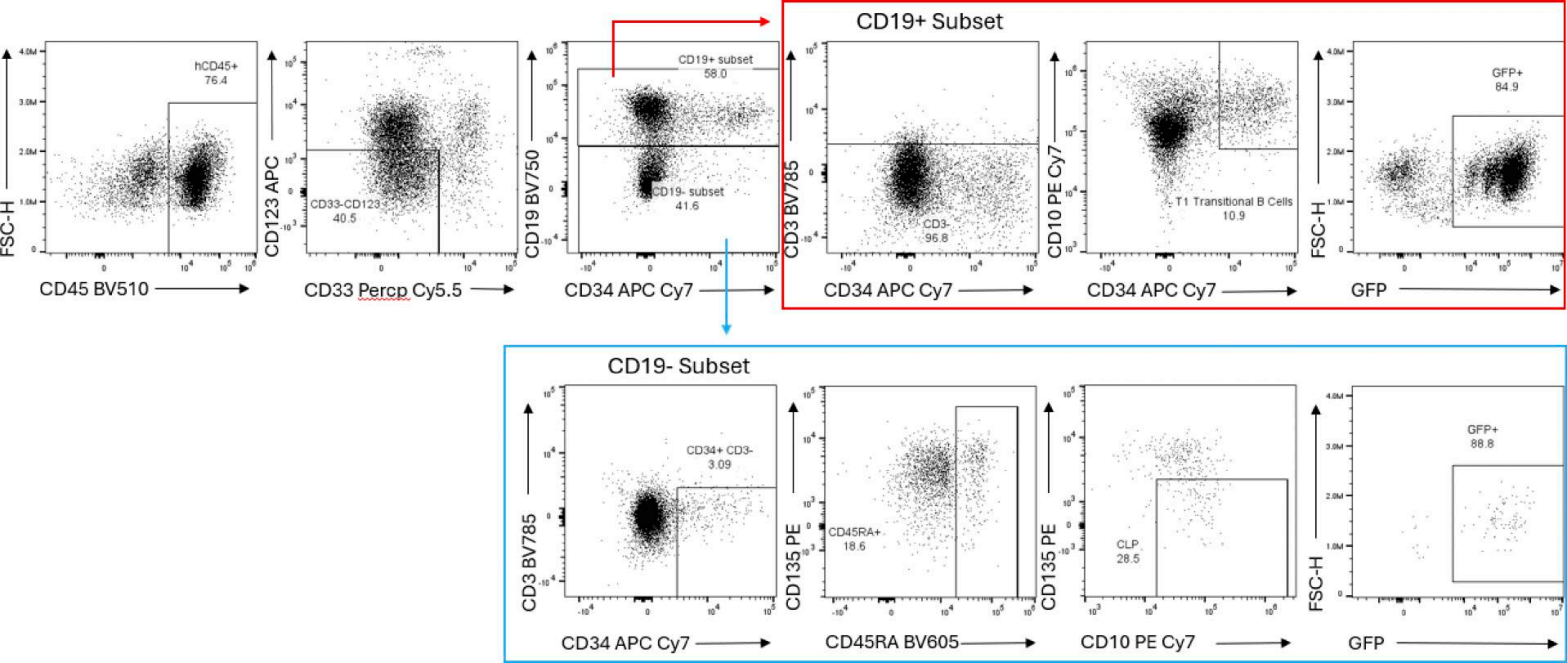
**Representative flow cytometry gating for xenograft BM samples.** The live singlet cells were gated for human CD45, and then, myeloid lineage was excluded (CD123^–^CD33^−^) prior to gating on CD19-negative (blue) or CD19-positive (red) populations. The CLP was further defined based upon CD19^−^CD3^−^CD34^+^CD10^+^ CD45RA^+^ CD135^−^ (CD38^+^; data not shown). The hematogones (early B cell progenitors) were first captured in CD19^+^ (red) population and then further gated based upon CD19^+^CD3^−^CD34^+^CD38^+^CD10 (CD38^+^; data not shown). Percent GFP was positive in each population.

To more formally test whether SEPTIN6 mutant stem cells could develop into B cells, we modeled the disease in the NOD.Cg-*Kit*^*W-41J*^*Tyr*+*Prkdc*^*scid*^ *Il2rg*^*tm1Wjl*^/ThomJ (NBSGW) xenograft mouse model (13). First, we created a bidirectional lentiviral vector (LV) expressing a SEPTIN6 wild-type (WT) or stop-loss plus extension mutant protein (*428Qext*9) genetic mutant (Fig. 4 E) (5). The construct encoded a GFP reporter with cis-linked pri-miRNA–based RNA interference (short hairpin miRNA [shmiR]) to silence the endogenous *SEPTIN6* (14). Donor CD34^+^ HSCs (*n* = 7 donors) were transduced with the SEPTIN6-expressing LV, or an empty vector with comparable transduction efficiency. These cells were then transferred into NBSGW recipients, and peripheral blood was monitored for 4 mo during engraftment prior to BM analysis. We focused on BM lymphoid populations defined as CLPs (Lin^−^CD34^+^CD38^+^CD19^−^CD10^+^CD45RA+CD135^−^) and transitional B cells (Lin^−^CD34^+^CD38^+^CD19^+^CD10^+^) (Fig. S3). NBSGW animals received cells in two separate experiments, and the percentages of transduced donor cells (GFP+) in the CLP or transitional B cell populations at 4 mo after transfer were pooled for analysis (Fig. 4 F). Animals with fewer than 100 cellular flow events for either population were excluded from the analysis. Overall, we found a significantly reduced percentage of GFP+ in the CLP population when mutant SEPTIN6 was expressed (*n* = 6 recipients) compared with empty vector or WT controls (*n* = 9 recipients). Similarly, transitional B cells expressing mutant SEPTIN6 (*n* = 8 recipients) showed a reduced percentage of GFP+ compared with empty vector or WT transduced cells (*n* = 9 recipients). Therefore, in the xenograft model, human CD34^+^ cells expressing a SEPTIN6 stop-loss variant did not show a B cell development block, but instead a reduction in lymphoid progenitors.

### SEPTIN6-related immunodeficiency is ameliorated with myeloablative HSCT

Lastly, we report the successful treatment of SEPTIN6-related disease with both affected siblings undergoing myeloablative HSCT. Similar to the first reported case (5), the proband (III.d) reported here had full reversal of symptoms following myeloablative HSCT including full donor chimerism at 12 mo after HSCT (previously mixed only in T cell lineage) and a posttransplant course only notable to severe mucositis. His brother (III.e) has engrafted with 100% chimerism to date. Both siblings received the same 10/10 HLA-matched sibling donor cryopreserved BM grafts. HSCT for the proband (III.d) was performed at ∼10 mo of age with myeloablative conditioning including busulfan (area under the curve [AUC] of 90 mg*hr/L) and fludarabine (160 mg/m2 i.v. given over 4 days). The GVHD prophylaxis included cyclosporine starting on day −1 and methotrexate on days +1, +3, and +6. Day 11 methotrexate was omitted due to severe mucositis. The proband engrafted neutrophils on day +17 and platelets on day +40. He had full donor myeloid chimerism and mixed donor T cell chimerism until achieving full chimerism at 12 mo after HSCT. The patient discontinued immunoglobulin replacement therapy with stable immunoglobulin levels (∼17 mo after HSCT) with protective antibody responses to both inactive and live vaccinations. In the proband, there was no acute or chronic GVHD disease 3 years after HSCT. Chimerism continued to be 100% donor in all lineages (CD3^+^, CD33^+^, CD56^+^, CD19^+^, CD14^+^ cells) more than 3 years after transplant.

The younger brother (III.e) underwent HSCT at 5 mo of age, using myeloablative conditioning regimen with busulfan (AUC 85 mg*hr/L) and fludarabine 5.32 mg/kg given over 4 days followed by a washed, cryopreserved, and matched BM graft. The GVHD prophylaxis included cyclosporine (switched to tacrolimus due to electrolyte abnormalities around day +42) and mycophenolate mofetil, which was substituted for methotrexate due to hepatic dysfunction. He engrafted neutrophils on day +21 and platelets on day +28. His posttransplant course was complicated by grade IIb acute skin GVHD on day +24 treated with topical and systemic steroids with complete response and subsequent taper. BM evaluations on day +42 and day +100 demonstrated mildly hypocellular marrow with normal cytogenetics and full donor chimerism. Peripheral blood lineage–specific chimerism has demonstrated full myeloid donor chimerism, with high donor followed by full donor T cell chimerism as of day +100 after transplant. He remains on immunoglobulin replacement at 5 mo after transplantation. In both patients, HSCT fully reversed the underlying inborn error of immunity (IEI) due to SEPTIN6 variants.

## Discussion

One powerful tool to understand hematopoiesis is to study patients with monogenic IEI–related cytopenias. Here, we present the second report on X-linked SEPTIN6-related immunodeficiency observed in two affected siblings with nonsyndromic severe peripheral neutropenia, tetraploid myeloid cells, and predisposition to aneuploidy. In our study, we describe several key new findings: first, we report a novel germline *SEPTIN6* stop-loss mutation resulting in a C-terminal protein extension. The extension likely disrupts protein function as the C terminus is critical for complex filament formation involving septin proteins (15). Second, while the initial case was a de novo variant and predicted to be dominant, we identified an asymptomatic maternal carrier of the pathogenic allele. Our data show that maternal peripheral blood exclusively expressed the unmutated allele. *SEPTIN6* has been shown to escape X-chromosome inactivation in both spleen and whole blood samples in healthy females (16). Therefore, we hypothesize a strong negative selection at the level of stem cells expressing the *SEPTIN6* stop-loss variants. Third, we identified a profound lack of circulating B cells and early B cell progenitors (hematogones) in the BM of both siblings, a finding also found retrospectively in the initial case as well (5). Fourth, transcriptional data and xenograft modeling showed a role of SEPTIN6 in generating lymphoid-skewed and early B cell progenitors. Collectively, our data support the pathogenicity of stop-loss variants in the *SEPTIN6* gene causing an IEI with the rare combination of severe congenital neutropenia, B cell deficiency, and non-SCID T cell lymphopenia.

In the cases presented here, both affected siblings were positive for an abnormal SCID NBS. The TREC testing showed counts as low as zero, but confirmatory flow cytometry testing showed naive (CD45RA^+^CD45RO^−^) CD4^+^ and CD8^+^ T cells were present in normal proportions in both affected males highlighting adequate thymic function. The T cell compartment eventually normalized in the proband, but T cell lymphopenia was more persistent in the younger brother. Similar to other non-SCID T cell lymphopenia identified by NBS, including several BM production defects, variable and delayed recovery for the T cell lymphopenia was observed (17). Here, we modeled human lymphoid development in immunodeficient mice and found the SEPTIN6 stop-loss variant caused reduced common lymphoid and early B cell progenitors compared with untransduced cells in the same xenograft host suggesting a cell-intrinsic effect. Unfortunately, the NBSGW model does not efficiently support T cell development, so we cannot further comment on T cell phenotypes. Individuals with SEPTIN6-related disease have been shown to have reduced fitness of HSCs and myeloid progenitors (5). Our data highlight a role of SEPTIN6 also in lymphopoiesis, but further studies are needed to define this in each lymphocyte population.

In this study, we focus on persistent B cell lymphopenia and absent early B cell progenitors in patients with SEPTIN6 stop-loss variants. Despite low progenitor and mature B cells, we also observe plasma cells in the marrow and detect immunoglobulin production. Our xenograft data showed reduced B cell progenitors rather than an absolute block in B cell development. This phenotype resembles GATA2 deficiency with reduced hematogones and peripheral B cell lymphopenia despite abundant plasma cells (18). GATA2 haploinsufficiency also results in defective cytokinesis and risk for aneuploidy (19). However, unlike SEPTIN6-related disease, GATA2 haploinsufficiency often displays underdifferentiated myeloid cells including monocytopenia, low dendritic cells, and neutrophils that are hypogranular and have pelgeroid features (20). More akin to SEPTIN6-related disease, germline heterozygous LCP1 variants cause an immunodeficiency with G-CSF–resistant congenital neutropenia, lymphopenia, and tetraploid hematopoiesis including hyperlobated myeloid forms (21, 22, 23). However, detailed analysis of B cells was not performed. Other gain-of-function variants in genes regulating actin dynamics have also been reported with the combined phenotype of tetraploidy and neutropenia; however, the lymphocyte phenotypes are either not reported or milder (24, 25). Future studies with SEPTIN6 variant HSCs will focus on clarifying the lymphoid potential at distinct early progenitor stages compared with controls. Comparing the shared features and subtle differences of these IEI cytokinesis defects may provide a powerful tool to understand hematopoiesis defects.

SEPTIN6 functions within hetero-oligomeric scaffolds that aid in cytokinesis, cell motility, and membrane remodeling. We observed congenital neutropenia without a maturation arrest, yet hyperlobation and large cell size. The marrow lacked histologic signs of apoptotic debris and was also not hypercellular, but neither are sensitive measures. In our cases, there was also not an inflammatory environment skewing the marrow development. Therefore, we hypothesize that the SEPTIN6-related neutropenia may result from enlarged and hyperlobated cells becoming physically stressed or dying during egress from the marrow due to the cytoskeletal defect. Future studies could test whether introducing SEPTIN6 pathogenic variants causes a cellular migration defect or alternatively a survival defect as measured by apoptosis assays (e.g., Annexin V, cleaved caspase-3). Importantly, these studies could then be expanded to other IEI cytokinesis defects to understand the generality of the findings.

### Conclusion

Much remains unknown about the role of septins in humans, which may differ from murine studies, specifically in SEPTIN6-related diseases. Here, we reported the second and third case of a *SEPTIN6* germline defect, identifying a unique variant in male siblings with congenital neutropenia, B cell aplasia, and variable T cell lymphopenia successfully treated with HSCT. While remarkably similar to the initial report of SEPTIN6-related disease, our studies characterize the lymphocyte defects associated with SEPTIN6 stop-loss variants.

## Materials and methods

### Human subjects

All individual participants and/or parents gave their written informed consent under the Immunology Biorepository #667 protocols approved by Seattle Children’s Hospital Institutional Review Board including blood draws for standard peripheral blood mononuclear cell (PBMC) isolation, genomic sequencing, and marrow transcriptomics analysis. BM aspirates and biopsies were performed; the latter were formalin-fixed and paraffin-embedded (FFPE) according to standard clinical practices.

### Genomic and spatial sequencing

Variants for proband and parents were identified via whole-genome sequencing on whole blood performed at Rady Children’s Institute for Genomic Medicine via the clinical pipeline. Following negative results, fastq files were released for research analysis, including manual curation of .bam files using the Integrative Genomics Viewer (26). PBMC-derived genomic DNA or mRNA was isolated by genomic DNeasy or RNeasy kits (Qiagen) prior to cDNA synthesis kit (Qiagen) or directly to Sanger sequencing for validation. Targeted variant testing via Sanger sequencing was performed for maternal grandparents and siblings.

Slides were prepared using sections from the proband’s clinical BM biopsy FFPE blocks and were deparaffinized and prepared per the standard protocol (MAN-10150-01; NanoString). Control biopsy FFPE block was from an age-matched male with new diagnosis of untreated neuroblastoma with undetectable (<1%) involvement of the marrow. Adjacent sections were stained with RNAscope (ACD) 3-plex positive control housekeeping gene in situ hybridization probes to indicate RNA integrity (RNAscope v2 Intro Kit; ACD). GeoMx Digital Spatial Profiler (DSP) slides with tissue were incubated with UV-cleavable human whole transcriptome atlas panel probe set followed by hybridization probes for DNA (SYTO13-488), CD68 (Opal 570), and CD45/PTPRC (Opal 690) per the manufacturer’s protocol (ACD). Slides were loaded in GeoMx DSP, and ROIs (*n* = 47/sample) were selected for high cellularity, and oligonucleotide tags were released from each ROI and collected into standard 96-well plates. Tags were enumerated via next-generation sequencing (NGS), and the library was prepared from pooled wells per protocol (MAN-10153-01; NanoString). Illumina NGS (3.25 billion read pairs) was generated on a NovaSeq S4 sequencer (Novogene). Data analysis was performed using GeoMxAnalysisWorkflow 0.1.2 (standR) and the limma-voom pipeline (27).

### Xenotransplantation studies

All mouse experiments were approved by the Boston Children’s Hospital Institutional Animal Care and Use Committee. Human CD34^+^ cells were lentivirally transduced with a LV engineered with a bidirectional promoter with (1) phosphoglycerate kinase (PGK) promoter driving eGFP reporter cis-linked with a shmiR for *SEPTIN6*, and (2) the minimal CMV promoter driving full-length WT or mutant SEPTIN6 isoforms, both modified to be resistant to the shmiR. Transduced cells were cultured in serum-free expansion media II (STEMCELL Technologies) supplemented with 1% penicillin/streptomycin, 100 ng/ul stem cell factor (SCF), 100 ng/ul Flt3, 50 ng/ul thrombopoietin (TPO), 0.75uM SR1, and 35 nM UM171 (PeproTech). 2 days after transduction, 1 million cells were injected retro-orbitally into NBSGW mice (The Jackson Laboratory). Chimerism was assessed monthly for 4 mo after which BM was isolated and used for flow cytometry analysis. Cell populations were excluded if <100 cells were present in the immunophenotypically defined BM populations. CD34^+^ cells from four donors were used for experiment 1 and from three donors for experiment 2.

### Cell staining and flow cytometry

BM isolated cells were stained with antibodies obtained from BioLegend including anti-CD10-PE-Cy7 (clone HI10a), CD33-PerCP-Cy5.5 (WM53), CD34-APC-Cy7 (581), CD38-BV711 (HIT2), CD45RA-BV605 (HI100), CD49f-PacBlue (GoH3), CD90-AF700 (5E10), CD123-APC (6H6), CD133-PE-Dazzle (S16015F), CD45-BV510 (HI30), CD19-BV750 (HIB19), CD135-PE (BV10A4H2), CD3-BV785 (OKT3), and CD14-BV650 (M5E2). Cells were washed and acquired on a Cytek Aurora (CytekBio). Flow cytometry data were analyzed on FlowJo (BD Biosciences).

### Measurement of human IFN proteins

Cytokines were measured using Human ProInflammatory-9 S-Plex Ultra-Sensitive Kit (K15396S), IFN-α2α S-Plex Ultra-Sensitive Kit (K151P3S), and IFN-β S-Plex Ultra-Sensitive Kit (K151ADRS) from Meso Scale Diagnostics as described (bioRxiv).

### Statistics

Progenitor population analysis was performed in Prism 10.4.1 using an unpaired *t* test.

### Online supplemental material

The provided supplemental tables and figures include detailed information of both patient’s BM biopsies, as well as Complete Blood Count (CBC) data collected at the time of BM sampling (Tables S1 and S2). Table S3 shows IFN cytokine measurements for each patient and for the mother. Fig. S1 shows dysmyelopoiesis in peripheral blood and BM of patient III.d at 4 mo of age. Fig. S2 shows the presence of morphologically normal neutrophils in the BM. Fig. S3 shows flow cytometry of xenograft BM samples, completed to demonstrate the lack of an absolute block in B cell development in the presence of SEPTIN6 mutations.

## Supplementary Material

Table S1 shows detailed BM morphology, cytogenetics, and flow cytometry findings for patient III.d.

Table S2 shows detailed BM morphology, cytogenetics, and flow cytometry findings for patient III.e.

Table S3 shows cytokine measurement for patients II.b, III.d, and III.e.

## Data Availability

The data are available from the corresponding author upon reasonable request.
