# Supplementary material for: X-linked SEPTIN6-related congenital neutropenia and B cell deficiency
Source: J Hum Immun. 2026 May 4;2(4):e20250173. doi: 10.70962/jhi.20250173 (PMC13137943; doi:10.70962/jhi.20250173)
Supplement: Table S1 — shows detailed BM morphology, cytogenetics, and flow cytometry findings for patient III.d. [file jhi_20250173_tables1.docx]

**Supplemental Table 1. Detailed bone marrow morphology, cytogenetics and flow cytometry findings for patient III.d.**

| Age | Specimen | Morphology / Results |
| --- | --- | --- |
| 2 months | Complete blood Count (CBC) data | WBC: 1.52K/uL, RBC: 3.95MIL/uL, Hgb: 11.5 g/dL, Hct: 34.0%, MCV: 86.1 fL, Plt: 376 K/uL; RDW: 13.6%.  Manual Differential: Seg neutrophils: 13%; Lymphocytes: 75%; Monocytes: 10%; Eosinophils: 1%; Metamyelocyte: 1%; ABS Neut: 0.20 K/uL; ABS lymphs: 1.14 K/uL; ABS Mono: 0.15 K/uL |
|  | Peripheral blood smear | Red blood cells are normocytic, normochromic and appear normal in number without significant anisopoikilocytosis. Platelets are normal in number and morphology. White blood cells appear decreased with absolute neutropenia and lymphopenia. A subset of the mature neutrophils has increased nuclear lobation (>5 nuclear lobes). Occasional monocytes and eosinophils also have increased nuclear lobes. No circulating blasts are identified. |
|  | BM aspirate smear | Aparticulate aspirate smears with hypersegmented neutrophils, eosinophils, and monocytes. Decreased lymphocytes (including hematogones) for age. Myeloids 61%; Erythroids 34.2%, Blasts 0.8%, Lymphocytes 4.0% |
|  | BM core biopsy | Cartilage and tangential sampling of normocellular marrow (>95% cellularity) showing trilineage hematopoiesis. Only rare CD20+ and PAX5+ positive B-cells present. |
|  | BM cytogenetics | G-banded Karyotype: 92,XXYY[16]/46,XY[4] (total cells = 20) |
| 4 months on G-CSF | CBC | WBC: 3.5 K/uL; RBC: 3.89 MIL/uL; HGB: 10.3 g/dL; HCT: 31.0%; MCV: 79.7 fL; PLT: 185 K/uL; RDW: 20.5%.  Manual Differential: Seg neutrophils: 2%; Lymphocytes: 93%; Monocytes: 3%; Eosinophils: 1%; Basophils: 1%; ABS Neut: 0.19 K/uL; ABS lymphs: 3.26 K/uL; ABS Mono: 0.11 K/uL |
|  | Peripheral blood smear | Red blood cells are mildly decreased, normochromic, normocytic, and with mild to moderate anisopoikilocytosis including tear drop cells and scattered schistocytes; polychromasia is not increased. Platelets are normal in number and in appearance. White blood cells are decreased with a marked absolute neutropenia.  Neutrophils have increased nuclear lobes (>5), including some giant forms at the bottom of the smear with >8 lobes.  Rare left-shifted myeloid cells are present.  Monocytes also often demonstrate increased nuclear lobation. No blasts are identified. |
|  | BM aspirate smears | Normocellular aspirate smears with relatively decreased myeloid precursors with marked left-shifted maturation and marked morphologic changes including extremely enlarged cells throughout maturation (2->5 times the normal size of the cells). These cells have increased nuclear lobation even at young stages, with mature neutrophils showing between 5 and >15 lobes. Some of these more mature forms have long and thin chromatin filaments between lobes; cytoplasmic vacuoles are not appreciated. Eosinophils also are enlarged with scattered hypersegmented forms. Erythroid precursors are relatively increased without significant dysplastic features. Megakaryocytes are normal to mildly increased in number. Lymphocytes are decreased for age. No ring sideroblasts on iron stain. Myeloids 55.5%; Erythroids 41%, Blasts 1.0%, Lymphocytes 2.5% |
|  | BM core biopsy | Normocellular marrow with normal M:E ratio. Myeloid cells have increased nuclear lobation. Lymphocytes are decreased for age. Mild reticulin fibrosis (MF-1) is appreciated. Only very rare CD20+ and PAX5+ positive B-cells; CD79a highlights scattered plasma cells (overall <1% but focally up to 5%) |
|  | BM flow cytometry | No abnormal myeloid blast population identified |
|  | BM cytogenetics | G-banded Karyotype: 92,XXYY[11]/46,XY[9] (total cells = 20) MDS FISH panel did not identify any evidence of deletion 5q31, monosomy 7, deletion 7q31, trisomy 8 or deletion 20q); however, 43% to 59% of cells showed signal patterns consistent with tetraploidy. |
| 7 months | CBC | WBC: 2.31 K/uL; RBC: 4.72 MIL/uL; HGB: 9.6 g/dL; HCT: 31.3%; MCV: 66.3 fL; PLT: 258 K/uL; RDW: 20.7%.  Differential: Neut: 13.1%; Lym: 81.8%; Mono: 1.7%; Eos: 0.4%; Baso: 2.6%; Imm Grans: 0.4%; ABS Neut: 0.30 K/uL; ABS lymphs: 1.89 K/uL; ABS Mono: 0.04 K/uL |
|  | Peripheral blood smear | Red blood cells are mildly decreased, mildly hypochromic, microcytic, and with mild to moderate anisopoikilocytosis including target cells and tear drop cells; polychromasia is not increased. Platelets are normal in number and in appearance; scattered larger forms are present. White blood cells are decreased with an absolute neutropenia. Neutrophils have increased nuclear lobes (>5). No blasts are identified. |
|  | BM aspirate smears | Hypocellular aspirate smears with relatively markedly decreased myeloid precursors with full spectrum maturation. Many of the maturing myeloid cells are enlarged and include binucleate promyelocytes and myelocytes. Erythroid precursors are relatively markedly increased without significant dysplastic features. Megakaryocytes are relatively increased and include smaller forms and/or forms with hypolobated nuclei. Lymphocytes are decreased for patient’s young age. Myeloids 11%; Erythroids 86%, Blasts 0.3%, Lymphocytes 2.8%. |
|  | BM core biopsy | Variably cellular, but overall mildly hypocellular (60-70%) marrow for age with relatively decreased myeloid precursors; clusters of enlarged precursors are noted. Erythroid precursors and megakaryocytes are relatively increased. No definitive increase in reticulin fibrosis. |
|  | BM flow cytometry | No abnormal myeloid blast population identified |
|  | BM cytogenetics | G-banded Karyotype: 47,XY,+8[5]/92,XXYY[4]/46,XY[11]. (total cells = 20) MDS FISH panel did not identify any evidence of deletion 5q31, monosomy 7/deletion 7q31, trisomy 8, or deletion 20q; however, 42% to 51% of cells showed signal patterns consistent with tetraploidy. |
| 9 months  pre-transplant | CBC | WBC: 3.3 K/uL; RBC: 5.04 MIL/uL; HGB: 10.0 g/dL; HCT: 33.5%; MCV: 66.5 fL; PLT: 382 K/uL; RDW: 25.8%.  Differential: Seg neutrophils: 2.5%; Lymphocytes: 96.7%; Monocytes: 0.8%; ABS Neut: 0.08 K/uL; ABS lymphs: 3.19 K/uL; ABS Mono: 0.03 K/uL |
|  | BM aspirate smears | Hypocellular aspirate smears with relatively decreased myeloid precursors with full spectrum maturation. Many of the maturing myeloid cells (~40%) are enlarged with abnormal hypersegmentation of both immature and mature forms. Erythroid precursors are relatively increased with some nuclear to cytoplasmic dyssynchrony and occasional markedly enlarged forms. Megakaryocytes are normal in number but with occasional hypolobated forms seen. Myeloids 31.8%; Erythroids 47%, Blasts 1.0%, Lymphocytes 20.2%. |
|  | BM core biopsy | Variably cellular, but overall hypocellular (~50%) marrow for age with relatively decreased myeloid precursors and megakaryocytes. No increase in reticulin fibrosis. Markedly decreased PAX5+/CD20+ B-cells (<<1%) and only rare CD79a+ cells (<1%, suggestive of plasma cells by morphology). |
|  | BM flow cytometry | Mild immunophenotypic abnormalities identified among the myeloid blasts (0.61%) including increased HLA-DR expression. No abnormal B cell population identified. |
|  | BM cytogenetics | G-banded Karyotype: 47,XY,+8[6]/92,XXYY[4]/46,XY[10] (total cells = 20). MDS FISH panel identified trisomy 8 in 9% of nuclei. There was no evidence of deletion 5q31, monosomy 7/deletion 7q, or deletion 20q); however, 30% to-42% of cells showed signal patterns consistent with tetraploidy. |
|  | BM NGS testing | No clinically significant variants were detected in genes tested on University of Washington Myeloid Gene Panel by NGS (https://testguide.labmed.uw.edu/view/HCAPMY) |
| 12.5 months  day +82 post- transplant | CBC | WBC: 4.9 K/uL; RBC: 2.7 MIL/uL; HGB: 8.3 g/dL; HCT: 24.5%; MCV: 90.7 fL; PLT: 188 K/uL; RDW: 15.1%.  Differential: Seg neutrophils: 80.6%; Lymphocytes: 8.8%; Monocytes: 9.0%; Eosinophils: 0.8%; Basophils: 0.4%; Imm Grans: 0.4%; ABS Neut: 3.95 K/uL; ABS lymphs: 0.43 K/uL; ABS Mono: 0.04 K/uL |
|  | BM aspirate smears | Mildly hypocellular aspirate smears with relatively normal myeloid precursors, erythroid precursors, and megakaryocytes. No significant dysplastic features. Myeloids 58.5%; Erythroids 28%, Blasts 1.0%, Lymphocytes 12.5%. |
|  | BM core biopsy | Variably cellular, but overall hypocellular (~60%) marrow for age with relatively normal myeloid-to-erythroid ratio and megakaryocytes. |
|  | BM flow cytometry | No abnormal myeloid blast population or abnormal B- or T-cell population G-banded Karyotype: //46,XX[20] (consistent with donor origin) (total cells = 20)  MDS FISH panel did not identify any evidence of deletion 5q31, monosomy 7/deletion 7q31, trisomy 8, or deletion 20q |
|  | BM cytogenetics/FISH | G-banded Karyotype: //46,XX[20] (consistent with donor origin) (total cells = 20)  MDS FISH panel did not identify any evidence of deletion 5q31, monosomy 7/deletion 7q31, trisomy 8, or deletion 20q |
| 21 months,  11.5 months post- transplant | CBC | WBC: 5.9 K/uL; RBC: 4.04 MIL/uL; HGB: 11.0 g/dL; HCT: 32.7%; MCV: 80.9 fL; PLT: 315 K/uL; RDW: 12.8%.  Differential: Seg neutrophils: 58.1%; Lymphocytes: 32.4%; Monocytes: 6.2%; Eosinophils: 2.7%; Basophils: 0.3%; Imm Grans: 0.3%; ABS Neut: 3.43 K/uL; ABS lymphs: 1.91 K/uL; ABS Mono: 0.37 K/uL |
|  | BM aspirate smears | Normocellular aspirate smears with relatively increased myeloid precursors with full maturation. Erythroid precursors are relatively decreased. Megakaryocytes are normal in numbers. No significant dysplastic features. Myeloids 68.2%; Erythroids 16%, Blasts 0.6%, Lymphocytes 15.2%. |
|  | BM core biopsy | Normocellular (90-95%) marrow for age with relatively increased myeloid precursors, decreased erythroid precursors, and normal megakaryocyte numbers. |
|  | BM flow cytometry | No abnormal myeloid blast population or abnormal B- or T-cell population |
|  | BM cytogenetics | G-banded Karyotype: //46,XX[20] (consistent with donor origin) (total cells = 20)  MDS FISH panel did not identify any evidence of deletion 5q31, monosomy 7/deletion 7q, trisomy 8, and deletion 20q |
