# Supplementary material for: X-linked SEPTIN6-related congenital neutropenia and B cell deficiency
Source: J Hum Immun. 2026 May 4;2(4):e20250173. doi: 10.70962/jhi.20250173 (PMC13137943; doi:10.70962/jhi.20250173)
Supplement: Table S2 — shows detailed BM morphology, cytogenetics, and flow cytometry findings for patient III.e. [file jhi_20250173_tables2.docx]

**Supplemental Table 2**: **Detailed bone marrow morphology, cytogenetics, and flow cytometry findings for patient III.e**

| Age | Specimen | Morphology / Results |
| --- | --- | --- |
| 4 months | Complete Blood Count (CBC) data | WBC: 0.5 K/uL, RBC: 3.36 MIL/uL, Hgb: 8.7 g/dL, Hct: 26.8%, MCV: 79.8 fL, Plt: 189 K/uL; RDW: 16.4%.  Differential: Seg neutrophils: 4.5%; Lymphocytes: 91.1%; Monocytes: 0%; Eosinophils: 0%; Basophils: 2.2%; Immature granulocytes: 2.2%; ABS Neut: 23 K/uL; ABS Lymphs: 456 K/uL; ABS Mono: 0 K/uL |
|  | BM aspirate smear | Aparticulate aspirate smears with decreased and markedly left shifted myeloid cells. Occasional mature neutrophils are present (including rare forms with increased nuclear lobes). Subset of immature myeloid forms appear increased in size. Erythroid precursors are relatively increased and show increased mitotic activity. Megakaryocytes are present, but not well-represented in aparticulate aspirate material.  Manual Differential: Myeloids: 10.4%; Erythroids: 74.8%; Blasts: 1.2%; Lymphocytes: 12.8%; Plasmacytoid cells: 0.8% |
|  | BM core biopsy | Normocellular (90-95%) BM for age with decreased myeloid precursors, increased erythroid precursors and increased megakaryocytes.  CD79a is negative. |
|  | BM cytogenetics | G-banded Karyotype: 47,XY,+8[8]/48,XY,+8,+9[4]/92,XXYY[3]/45,XY,-7[3] /46,XY[9] (total cells = 27)  MDS FISH panel was positive for monosomy 7 in 7% of cells, positive for trisomy 8 in 12% of cells, and did not identify any evidence of deletion of 5q31 or deletion 20q; however, 40.6% to 49.6% of cells showed signal patterns consistent with tetrasomy. |
