# Supplementary material for: X-linked SEPTIN6-related congenital neutropenia and B cell deficiency
Source: J Hum Immun. 2026 May 4;2(4):e20250173. doi: 10.70962/jhi.20250173 (PMC13137943; doi:10.70962/jhi.20250173)
Supplement: Table S3 — shows cytokine measurement for patients II.b, III.d, and III.e. [file jhi_20250173_tables3.docx]

**Supplemental Table 3: Cytokine measurement for patients II.b, III.d and III.e.**

| Subject | Disease State | Age at time of sample | IFN-α^**^ | IFN-β^#^ | IFN-γ^$^ |
| --- | --- | --- | --- | --- | --- |
| III.d (proband) | Septin6-deficiency | 9 months | 5.6* | 15.4* | 108.5 |
| III.e | Septin6-deficiency | 1 day | 11.7* | 119.9 | 110.3 |
| II.b (mother) | Septin6 carrier | 32 years | 8.6* | 10.6* | 135.2 |

**Supplemental Table 3: Interferon levels in plasma.** Cytokines were all measured in femtograms (fg)/mL from plasma collected from whole blood at age indicated and measured via the Meso Scale Discovery platform. Lower limit of detection (LLOD) was defined as the concentration of diluted standard that provides signal 2.5 standard deviations above the mean of the blank value. Lower limit of quantification (LLOQ) was defined as the lowest concentration of diluted standard with a coefficient of variation (CV) less than 20% between replicates. Normal median and ranges below for S-PLEX Human IFN-α2a, -β or Proinflammatory Panel 1 kit (available at www.mesoscale.com).

* values lower than LLOD/LLOQ

** IFN-α: LLOD 18.7; LLOQ 47.8 fg/mL; normal sodium heparin plasma median 7.6 fg/mL;range (ND-260)

# IFN-β LLOD 7.8, LLOQ 73.3 fg/mL; normal sodium heparin plasma median 81 fg/mL;range (ND-59)

$ IFN-γ LLOD 7.8, LLOQ 73.3 fg/mL; normal sodium heparin plasma median 170 fg/mL; range (36-2100)
